# Family effects on the rurality of GP’s work location: a longitudinal panel study

**DOI:** 10.1186/s12960-017-0250-z

**Published:** 2017-10-19

**Authors:** Matthew R. McGrail, Deborah J. Russell, Belinda G. O’Sullivan

**Affiliations:** 1 Monash Rural Health, Monash University, Northways Road, Churchill, VIC 3842 Australia; 2 Monash Rural Health, Monash University, 26 Mercy Street, Bendigo, VIC 3550 Australia

**Keywords:** General practitioners, Workforce, Rural, Retention, Education, Employment, Location, Non-professional, Family

## Abstract

**Background:**

Reduced opportunities for children’s schooling and spouse’s/partner’s employment are identified internationally as key barriers to general practitioners (GPs) working rurally. This paper aims to measure longitudinal associations between the rurality of GP work location and having (i) school-aged children and (ii) a spouse/partner in the workforce.

**Methods:**

Participants included 4377 GPs responding to at least two consecutive annual surveys of the *Medicine in Australia: Balancing Employment and Life* (MABEL) national longitudinal study between 2008 and 2014. The main outcome, GP work location, was categorised by remoteness and population size. Five sequential binary school-age groupings were defined according to whether a GP had no children, only preschool children (aged 0–4 years), at least one primary-school child (aged 5–11 years), at least one child in secondary school (aged 12–18 years), and all children older than secondary school (aged ≥ 19). Partner in the workforce was defined by whether a GP had a partner who was either currently working or looking for work, or not. Separate generalised estimating equation models, which aggregated consecutive annual observations per GP, tested associations between work location and (i) educational stages and (ii) partner employment, after adjusting for key covariates.

**Results:**

Male GPs with children in secondary school were significantly less likely to work rurally (inclusive of > 50 000 regional centres through to the smallest rural towns of < 5000) compared to male GPs with children in primary school. In contrast, female GPs’ locations were not significantly associated with the educational stage of their children. Having a partner in the workforce was not associated with work location for male GPs, whereas female GPs with a partner in the workforce were significantly less likely to work in smaller rural/remote communities (< 15 000 population).

**Conclusions:**

This is the first systematic, national-level longitudinal study showing that GP work location is related to key family needs which differ according to GP gender and educational stages of children. Such non-professional factors are likely to be dynamic across the GP’s lifespan and should be regularly reviewed as part of GP retention planning. This research supports investment in regional development for strong local secondary school and partner employment opportunities.

## Background

The recruitment and retention of a sufficient general practitioner (GP) workforce in rural and remote locations is an ongoing problem internationally [1–3]. There are many professional and non-professional factors contributing to rural medical workforce supply and distribution difficulties [4, 5]. Several strategies have been evaluated as improving the rural medical workforce supply with particular focus on medical student selection, rural medical education and postgraduate training exposures and a range of regulatory interventions requiring rural practice [6–8]. Research has also revealed that key professional issues for GPs either considering or remaining in rural practice include longer work hours, higher on-call participation, difficulty getting time off work, adequacy of remuneration, difficulty accessing ongoing medical education and additional challenges related to supplying services in more remote, sparsely populated areas [3, 8–10]. Less research and policy attention has focused on non-professional factors and their influence on decisions about where GPs work.

Two key Australian studies have explored associations between non-professional factors and GPs’ work location. The first showed strong associations between reduced opportunities for the GP’s spouse/partner and children and working in smaller towns, especially very small rural or remote towns [11]. The second also found associations between significantly reduced GP satisfaction with non-professional factors (social and leisure opportunities as well as partner employment and choice of schools) and working in smaller towns [12]. Other studies from Australia, Canada, and the United States consistently identify two non-professional factors as key barriers to rural GP practice: (i) fewer and less comprehensive school opportunities for the children of GPs, particularly at the secondary school level, and (ii) fewer employment options for the spouse/partner of GPs [3, 13–21].

There is an acknowledged gap in educational achievements by rural Australian students compared to metropolitan students, with a current national review underway to investigate the range, quality and accessibility of educational opportunities for children in rural areas [22]. Recent national performance data of reading ability among third year secondary-school students found that 23% (metropolitan), 13–16% (rural and regional) and only 3–11% (remote and very remote) achieved scores in the highest two bands [23]. Australia’s geography and sparsely distributed population means there can be difficulties attracting and retaining quality teachers with potential negative impacts on educational quality and outcomes [24, 25]. Both completion of secondary-level school and attainment of a university degree are much less common among rural populations. Australian Bureau of Statistics 2011 census data show that 55% of all metropolitan residents completed secondary school versus 36% of rural residents [26]. Similarly, 22% of metropolitan residents have completed a minimum bachelor-level degree versus 12% of rural residents. Additionally, many small towns have no secondary school, while others have small schools with fewer educational enrichment options. The quality of rural schools and universities is also often perceived to be poorer, and this can be problematic, especially for families wanting their children to receive the level and quality of education needed for competitive entry into university courses and to gain professional employment.

Regional areas frequently also experience slower rates of economic [27] and population growth relative to metropolitan areas: 10-year population growth data demonstrates Australia’s increasing urbanisation with metropolitan areas having a 1.9% annual growth compared with 1.2% in rural and regional areas and only 0.8% in remote areas [28], consistent with international trends [29]. Further, in regional areas, a smaller proportion of adults work in professional roles (16% compared with 24% in metropolitan areas), and in rural areas, fewer industries require employees with secondary school completion. For many dual-profession couples, the regional job market limits their inclusion, although this may vary depending on skills, interests and professional flexibility of the spouse/partner [30].

While GPs without partners and school-aged children have more autonomy over where they work [31], most doctors have a spouse/partner and school-aged children during their career and endeavour to accommodate the needs of their family with respect to decisions about where they live and work. Rural areas may offer a preferred lifestyle and location for raising children, with a reduced pace of living, less traffic and more affordable housing [32, 33]. However, this amenity may change over time, as children grow up or spouse/partner employment needs change. It is possible that the role of non-professional factors will be lower for GPs with higher rural work propensity [9, 16, 31, 34, 35]. The best way to explore the role of non-professional factors is through longitudinal research design, to account for the dynamic nature of education and employment issues over the lifespan.

However, published research to date has been limited by its cross-sectional design, with inherent limitations for differentiating how changes in children’s educational stages or in partner employment needs over time are associated with GP work location choices. Further, there has been little investigation of the moderating effect that GP gender may have on these associations. Such issues are important to explore by gender, given that Australia is experiencing a surge in the number of female doctors.

This study aims to measure longitudinal associations between the rurality of GPs’ work locations and two key non-professional factors, firstly having children at different educational stages and secondly having a partner/spouse in the workforce, investigating how these vary by gender.

## Methods

This research uses panel data from the large *Medicine in Australia: Balancing Employment and Life* (MABEL) study, conducted within the Centre for Research Excellence in Medical Workforce Dynamics. MABEL is an Australian national longitudinal survey, which collects annual data from a panel of doctors (http://mabel.org.au/). The first wave of MABEL, in 2008, invited the entire Australian medical workforce to participate, with 10 498 doctors (19.4% response rate) completing the initial survey, including 17.7% (3996) of Australia’s GPs. This was demonstrated to be a highly representative cohort [36]. Each year, respondents to the previous wave and new doctors (mostly newly graduating non-specialist hospital doctors or newly arrived international medical graduates (IMGs)) are invited to complete the MABEL survey. The panel survey has an annual 70–80% study retention rate of previous respondents and adding new doctors has maintained the total annual respondents at about 9000–10 000. This study uses data from waves 1 to 7 (2008 to 2014) and only includes general practitioners (GPs), with GP Registrars, who are completing their vocational training at the time of each survey, excluded from the study.

The outcome variable, main work location of each GP, was geocoded for each wave to a specific town or suburb, then classified using the Modified Monash Model (MMM) scale (Table 1), a robust geographic rurality indicator capturing both remoteness and town size, primarily adopted within Australian policy to determine eligibility for rural GP retention payments [37]. The MMM scale has seven levels, with MMM-1 defining all metropolitan locations, MMM-2 to MMM-5 defining all rural locations, separated by decreasing population size cut-offs of > 50 000; 15 000–50 000; 5000–15 000 and < 5000, while MMM-6 and MMM-7 (combined here due to small counts) define all remote and very remote locations. A key focus of this paper is to explore how the rurality of the GP’s work location varies according to the educational stage of the GP’s children and employment needs of partners, so a series of four binary rural location groups were defined based on the MMM scale. These were MMM-1 versus MMM 2–7 (any ‘rural’), 1–2 versus 3–7 (< 50K), 1–3 versus 4–7 (< 15K) and 1–4 versus 5–7 (< 5K and ‘remote’).

**Table 1:** The Modified Monash Model (MMM) rurality classification

Category	Label/definition	Type	Australia’s population (%)
MMM-1	Major cities	Metropolitan	70.0
MMM-2	Regional centres, > 50K	Rural	9.3
MMM-3	Large rural towns, > 15K and < 50K	Rural	6.7
MMM-4	Medium rural towns, > 5K and < 15K	Rural	3.8
MMM-5	Small rural towns, < 5K	Rural	7.9
MMM-6 + 7	Remote and very remote communities	Remote	2.3

K = 1000 population

To define educational stage, GPs were asked whether they had any dependent children and also the age of each child. This information was used to define sequential and non-overlapping binary groupings in each wave, enabling measurement of associations with a GP’s children transitioning to a different stage: (1) having no children; (2) having at least one child but all of preschool stage (aged 0–4 years); (3) having at least one child in primary school (aged 5–11 years), but none of secondary-school age; (4) having at least one child in secondary school (aged 12–18 years); and (5) having only children no longer at school (aged ≥ 19). Tested group comparisons were (2) versus (1), (3) versus (2), (4) versus (3) and (5) versus (4). Secondly, at each wave, GPs were asked if they currently lived with a partner/spouse (simplified to partner henceforth) and their partner’s employment status. The variable ‘partner in the workforce’ was defined in each wave using a binary grouping of ‘yes’ if the GP both had a partner and the partner was either currently working or looking for work and ‘no’ if these conditions did not apply.

### Statistical analysis

Separate generalised estimating equations (GEEs) were applied to explore the association between work location and the two key predictive variables of educational stage and partner employment, stratified by gender. GEE models specifically account for the high level of within-person correlation in repeated measures of longitudinal studies and enable the full dynamic nature of the predictive variables and GP work location at each wave to be captured. A logit link function and autoregressive (lag 1, or AR1) correlation structure were used, aggregating all dynamic observations per doctor over a period of up to 7 years, irrespective of whether doctors moved or stayed in the same location. Interpretation of this GEE model is similar to that of a logistic regression model. The AR1 structure, which assumes a strong correlation of outcome between consecutive periods but decays exponentially as the period gap increases, required at least two observations (excluding about 7% of responses) and a continuous period of observations (consecutive survey responses, further excluding about 4%). Given that respondents can come and go from participating in the MABEL survey, only the longest period of consecutive responses was included (excluding an additional 5%). Model adjustments were made for being an international medical graduate (IMG) and having a rural origin (≥ 6 years of childhood in a rural location), both of which have been independently associated with rural GP practice in previous research [38, 39]. All calculations were performed using StataSE 14 (StataCorp, Texas USA) with a 5% significance level.

## Results

A total of 4377 GPs completed at least two surveys in consecutive years, with the total eligible observations across seven waves being 18 333 (average 4.2 per GP). The demographic characteristics of the included respondents are summarised in Table 2. Notably, about 45% GPs had at least one school-aged child, while 30% had at least one child of secondary-school age and most (67%) had a partner in the workforce.

**Table 2:** Characteristics of GP respondents, MABEL survey, waves 1–7

Characteristic	*N* (%)^a^
MMM-1	11 878 (64.8)
MMM-2	1 888 (10.3)
MMM-3	1 531 (8.4)
MMM-4	963 (5.3)
MMM-5	1 222 (6.7)
MMM-6 + 7	851 (4.6)
Male	9 513 (51.9)
Female	8 810 (48.1)
Australian medical graduate	13 819 (75.6)
International medical graduate	4 466 (24.4)
Metropolitan origin (childhood)	11 576 (72.7)
Rural origin (childhood)	4 357 (27.4)
Spouse/partner in the workforce	11 944 (67.1)
No spouse/partner/not in the workforce	5 864 (32.9)
No children of any age	6 706 (37.0)
Oldest child of preschool age	1 048 (5.8)
Oldest child of primary-school age	2 597 (14.3)
At least 1 child of secondary-school age	5 496 (30.3)
All children above school age	2 275 (12.6)

Only includes MABEL participants who responded at least twice and in consecutive waves

^a^Missing values: MMM, Gender and IMG status each had 0–2% missing; Rural origin had 13% missing; Partner employment and Age of children had 4–6% missing

Table 3 summarises associations (odds ratios, ORs) between having children at different educational stages and GP work location. Notably, contrasting gender differences were found. Male GPs with only preschool children (versus no children) or at least one child in primary school (versus preschool) were similarly likely to be working rurally. However, male GPs with at least one child in secondary school (versus primary school) had consistently significantly lower odds of working in a rural location for all four rurality definitions (estimated ORs ranging between 0.83 and 0.90). Female GPs with children in either preschool or primary school consistently had reduced odds (less than 1) of working rurally; however, no associations were significant. The patterns of rural distribution for female GPs with preschool, primary-school and secondary-school children were similar.

**Table 3:** Odds of GP having children of (i) preschool age, (ii) primary-school age, (iii) secondary-school age and (iv) above school age and working rurally, stratified by gender

		MMM 2–7 v 1	MMM 3–7 v 1–2	MMM 4–7 v 1–3	MMM 5–7 v 1–4
	Male GPs				
(i) Oldest child aged 0–4	OR (95% CI)	1.07 (0.91, 1.26)	1.11 (0.91, 1.35)	1.18 (0.91, 1.54)	1.01 (0.74, 1.39)
(ii) Oldest child aged 5–11	OR (95% CI)	1.02 (0.89, 1.17)	1.04 (0.91, 1.18)	1.09 (0.94, 1.26)	1.05 (0.87, 1.27)
(iii) 1 or more child aged 12–18	OR (95% CI)	0.90* (0.83, 0.99)	0.83* (0.75, 0.92)	0.84* (0.74, 0.94)	0.83* (0.71, 0.96)
(iv) All children aged ≥ 19	OR (95% CI)	1.03 (0.95, 1.11)	1.04 (0.95, 1.13)	1.08 (0.97, 1.20)	1.03 (0.89, 1.20)
	Female GPs				
(i) Oldest child aged 0–4	OR (95% CI)	0.95 (0.77, 1.17)	1.00 (0.77, 1.29)	0.93 (0.66, 1.31)	0.82 (0.55, 1.23)
(ii) Oldest child aged 5–11	OR (95% CI)	0.92 (0.81, 1.06)	0.89 (0.75, 1.05)	0.92 (0.75, 1.12)	0.93 (0.70, 1.24)
(iii) 1 or more child aged 12–18	OR (95% CI)	1.01 (0.92, 1.11)	1.01 (0.89, 1.14)	1.01 (0.87, 1.18)	1.10 (0.90, 1.35)
(iv) All children aged ≥ 19	OR (95% CI)	0.99 (0.90, 1.08)	0.98 (0.88, 1.10)	0.95 (0.81, 1.11)	1.01 (0.81, 1.25)

The comparison groups are, in turn, having (i) no children, (ii) only preschool children, (iii) primary-school children but no secondary-school children and (iv) secondary-school children. All models included IMG status and childhood rural origin

*p<0.05

Table 4 summarises the association between having a partner in the workforce and GPs’ observed work locations, with key differences evident by gender. Male GPs, whether they had a partner in the workforce or not, were similarly likely to be working rurally (OR 1.00). In contrast, female GPs with a partner in the workforce were less likely to be working in smaller rural and remote communities (MMM 4–7: OR 0.89, *p* = 0.036) compared to female GPs without a partner in the workforce.

**Table 4:** Odds of GPs having a partner in the workforce and working rurally, stratified by gender

		MMM 2–7 v 1	MMM 3–7 v 1–2	MMM 4–7 v 1–3	MMM 5–7 v 1–4
Male GPs	OR (95% CI)	1.00 (0.96, 1.03)	1.01 (0.96, 1.05)	1.01 (0.96, 1.07)	0.98 (0.91, 1.05)
Female GPs	OR (95% CI)	0.95 (0.89, 1.01)	0.94 (0.87, 1.02)	0.89* (0.79, 0.99)	0.87 (0.75, 1.01)

All models included IMG status and childhood rural origin

*p<0.05

## Discussion

This is the first systematic, national-level longitudinal study showing that the rurality GP work location is related to family influences of schooling needs and partner employment, and the effect differs according to GP gender and the educational stage of the GP’s children. These data, drawn from a 7-year observation of Australian GPs, provide important clarification of the role of these two non-professional factors, which although widely recognised had not previously been quantified using rigorous longitudinal methods.

Female GPs with children in preschool and primary school were more likely to be located in metropolitan areas and regional centres compared to female GPs without children or with children in preschool, respectively; however, there was no association with work location for female GPs when their children reached secondary school. These findings suggest that work location decisions made by female GPs occur when their children are younger, whereby they are more likely to choose to live in larger regional centres or metropolitan locations, perhaps to enable better access to family and other supports, additionally enabling improved professional employment opportunities for their spouse/partner.

For male GPs, having children in secondary school was associated with increased odds of working in larger rural towns, regional centres or metropolitan locations. These findings confirm the broader literature that having children in secondary school significantly impacts location decisions of rural GPs and is important given that most rural GPs are male [1]. Further analysis of our data shows there was not a significant corresponding increase in choice of rural work once all of their children completed secondary school, suggesting the loss of male GPs across rural locations related to secondary-school needs are sustained long term, which is consistent with the reduced mobility of older GPs [40].

Having a partner in the workforce was not associated with work location for male GPs. The opposite was observed for female GPs with a partner in the workforce, who were significantly less likely to be located in smaller towns than female GPs without a partner in the workforce (ORs ranging between 0.88 and 0.94). It is possible that partners of female GPs have less flexible professional roles or specific skills and interests more suited to metropolitan or larger regions than partners of male GPs. In contrast, the partners of male GPs may be more flexible with regard to careers, skills and interests, thus supporting their ability to live in varied locations to fit with their GP partner’s career [41–44].

These findings suggest that for successful recruitment, distribution and long-term retention of rural GPs, it is important for rural workforce planners and employers to pay sufficient attention not only to meeting the professional needs of the GPs, but also to considering and meeting the changing educational and employment needs of the GP’s family which vary by gender and the ages of their children. The importance of these non-professional factors is likely to be very dynamic across the lifespan and should be regularly reviewed as part of GP recruitment and retention planning. People charged with recruiting GPs to rural communities might ensure that they have a good understanding of the strengths and limitations of their particular community with regard to preschool, primary-school and secondary-school educational opportunities as well as the range of employment options that might suit the GP’s spouse/partner. Assessment tools such as the Community Apgar Questionnaire can help identify a community’s relative advantages and challenges in this regard, compared with other similar communities [45]. Our research helps inform the importance of these factors for GPs by gender, spouse and family characteristics. Having this deeper level of understanding of the likely family needs of different GPs, in combination with the amenities of the community, may enable rural communities to better target their marketing and recruiting of GPs. Where a GP’s family’s needs can be met within existing community employment and educational infrastructure, this may, in turn, support longer retention.

In situations where educational and partner employment needs are unable to be sufficiently well-met, recruitment and retention may be supported by direct financial compensation in lieu of forgone employment and educational opportunities, although this needs to be incorporated as part of a more comprehensive retention package as the research suggests that financial incentives alone are unlikely to be effective [46–48]. With regard to education, high-achieving professionals often hold similar expectations for their family and their children, requiring high-quality educational opportunities, particularly in secondary school which governs most entries to university. It follows that the GP’s location choices are influenced by perceptions of the availability of high-quality schooling. This is achievable in regional areas and rural towns but often require specific planning and innovative thinking, such as flexible educational in- and out-reach programs, field trips and high-quality teachers and online resources. Other options may include private boarding school fees in employment packages or support for retraining into alternative employment fields for partners [49], though available evidence suggests these will only have limited impact and need to be bundled with other support mechanisms. Additionally, a number of small-scale strategies exist in Australia which support the families of rural doctors, such as partner retraining grants and formalised support networks [50–52]; there is no evidence of their impact on rural retention.

Investments in communication and technology infrastructure have the potential to better support partner employment opportunities and ensure regions are attractive to a professional workforce such as GPs [53]. Broader scale investments in regional development play a strong role in developing and diversifying regional jobs, building on community strengths and existing community infrastructure. The Australian government has a policy commitment to regional development and a current parliamentary inquiry into regional development and decentralisation initiatives, but there remains limited evidence about what works well specifically for schools and employment [54, 55].

Additionally, while this study has identified important and significant associations between observed work locations of GPs and the schooling needs of children or partner needs for employment, only a relatively small proportion of GPs were observed changing work location. This helps explain why none of the observed ORs were extreme, with the strongest associations having OR values around 0.80–0.85. This ‘moderate’ effect size is consistent with other literature which indicates that choice of work location involves complex decisions, balancing a multitude of professional and non-professional factors, all of which may only have a small to moderate contribution to the final decision [3, 5, 18, 56].

The limitations of our study include that it is not sensitive to additional factors which may have contributed to decisions of work location rurality made by GPs, either before or during the study period. This includes factors which were not captured by our analysis, such as those relating to restrictions on practice location or rural exposure during medical training. Additionally, the gender of the GP’s partner may also affect location choices; however, these data were not collected in the MABEL survey. Also, while location decisions may be influenced by opportunities for education and employment for family members, the timing of their impact was not possible to measure in our study. For example, GPs yet to have children can choose at the outset not to work rurally because, long term, based on future needs they will prefer to raise their family in a metropolitan setting. Finally, although this study demonstrates that family factors are related to GP location, more qualitative research about the reasons why employment or education factors influence different genders would be useful to pursue.

As a strength, our study was methodologically robust, using the GEE method to enable the full dynamic nature of the data to be utilised, accounting for key covariates. Our more finely grained approach helps to specify both *where* and *who* that family effects impact, thus better informing future policy directions. Furthermore, our study’s longitudinal panel design with full variability of both phenomena of interest and work location observed each year, the collection of data over a 7-year period, its national scale and this method of analysis optimise the statistical power of this study.

## Conclusion

Analysing the best available longitudinal data on individual Australian GPs, this landmark national-level study provides new empirical evidence of the significance of associations between the rurality of the work location of GPs and key non-professional factors. It shows that the educational stage of a GP’s children and having a partner in the workforce are associated with the rurality of a GP’s work location but associations differ according to GP gender. This evidence uniquely quantifies the small to moderate, yet significant, influence on GP work location choices of these two family considerations. These findings reinforce the importance of employers and recruitment agencies considering the family needs of GPs when recruiting and factoring in the dynamic changes in both education and employment needs of a GP’s family within retention planning, particularly in smaller towns. The findings more broadly support regional development investments to deliver high-quality local schooling and employment options in order to facilitate this key professional workforce.

## Abbreviations

AR: Autoregressive
GEE: Generalised estimating equation
GP: General practitioner
IMG: International medical graduate
MABEL: Medicine in Australia: Balancing Employment and Life
MMM: Modified Monash Model
OR: Odds ratio
